# NOX1 promotes myocardial fibrosis and cardiac dysfunction *via* activating the TLR2/NF-κB pathway in diabetic cardiomyopathy

**DOI:** 10.3389/fphar.2022.928762

**Published:** 2022-09-26

**Authors:** Dandan Zhang, Yilan Li, Weijie Wang, Xueyan Lang, Yanxiu Zhang, Qianqian Zhao, Jingru Yan, Yao Zhang

**Affiliations:** ^1^ Department of Cardiology, The Second Affiliated Hospital of Harbin Medical University, Harbin, China; ^2^ Key Laboratory of Myocardial Ischemia, Ministry of Education, Harbin Medical University, Harbin, China

**Keywords:** diabetic cardiomyopathy, Nox1, TLR2/NF-κB, myocardial fibrosis, oxidative stress, cardiac function

## Abstract

Diabetic cardiomyopathy (DCM) is a prevalent complication in patients with diabetes, resulting in high morbidity and mortality. However, the molecular mechanisms of diabetic cardiomyopathy have yet to be fully elucidated. In this study, we investigated a novel target, NOX1, an isoform of superoxide-producing NADPH oxidase with key functional involvement in the pathophysiology of DCM. The DCM rat model was established by a high-fat diet combined with streptozotocin injections. DCM rats elicited myocardial fibrosis exacerbation, which was accompanied by a marked elevation of NOX1 expression in cardiac tissue. In particular, a specific NOX1 inhibitor, ML171, effectively decreased myocardial fibrosis and protected against cardiac dysfunction in DCM rats. Rat neonatal cardiac fibroblasts were incubated with high glucose (HG, 33 mM) as an *in vitro* model of DCM. We also observed that the expression of NOX1 was upregulated in HG-cultured cardiac fibroblasts. Silencing of NOX1 was found to attenuate myocardial fibrosis and oxidative stress in HG-induced cardiac fibroblasts. Furthermore, the upregulation of NOX1 by hyperglycemia induced activation of the TLR2/NF-κB pathway both *in vitro* and *in vivo*, whereas these effects were significantly attenuated with NOX1 gene silencing and further enhanced with NOX1 gene overexpression. In summary, we demonstrated that NOX1 induced activation of the TLR2/NF-κB pathway and increased reactive oxygen species production accumulation, which ultimately increased myocardial fibrosis and deteriorated cardiac function in diabetic cardiomyopathy. Our study revealed that NOX1 was a potential therapeutic target for DCM.

## Introduction

Diabetic cardiomyopathy (DCM) is a prevalent complication of diabetes and a major cause of morbidity and mortality (Sun et al., 2020). DCM is a series of changes in the myocardial structure and function caused by diabetes that are not related to hypertension, coronary atherosclerosis, valvular heart disease, and other known heart diseases (Yang et al., 2020). However, DCM is the result of multiple etiologies, which are characterized by cardiac hypertrophy, myocardial fibrosis, and cardiomyocyte apoptosis (Fein, 1990; Huynh et al., 2014). Oxidative stress, cardiomyocyte metabolic disorder, mitochondrial dysfunction, advanced glycation end products, inflammation, and various other mechanisms are implicated in the progression of diabetic heart diseases (Anderson et al., 2011; Bugger and Abel, 2014; Jia et al., 2018). However, the underlying molecular mechanisms of DCM remain poorly defined. As a result, it is critical to investigate its molecular basis during the progression of DCM.

The NADPH oxidase (NOX) family is a primary source of aberrant reactive oxygen species (ROS) production in the cardiovascular system (Griendling et al., 2000; Panday et al., 2015). NOX1, NOX2, and NOX4 are three NOX isomers that are highly expressed in cardiac tissues (Lassegue et al., 2012). Previous studies have shown that NOX2 inhibition mitigates the harmful effects of hyperglycemia on cells (Balteau et al., 2011; Joseph et al., 2014), and inhibiting NOX2-induced oxidative stress could prevent DCM (Tang et al., 2019). In addition, NOX4 has been confirmed to reduce myocardial interstitial fibrosis in diabetic mice by regulating the Akt/mTOR and NF-κB pathways, thereby improving cardiac function (Zhao et al., 2015). NOX1 has been reported to be associated with a variety of cardiovascular diseases, mainly atherosclerosis, hypertension, and ischemia/reperfusion injury (Szocs et al., 2002; Dikalova et al., 2005; Niu et al., 2010; Braunersreuther et al., 2013; Sobey et al., 2015). Stephen et al. demonstrated that NOX1 is a potential therapeutic target in diabetic vasculopathies. They showed that deficiency of NOX1 reduces lesion size and ROS levels in the aorta in the presence of diabetes (Gray et al., 2013). Furthermore, several studies have revealed that NOX1 is an important factor in mediating oxidative stress and promoting fibrosis in other tissues (Paik et al., 2011; Zhu et al., 2015; Jung et al., 2020). However, the role of NOX1 in myocardial fibrosis in DCM has never been mentioned.

According to a previous study by Lee et al. (2013), toll-like receptor 2 (TLR2) has an interaction with NOX1 based on evidence from using yeast two-hybrid and co-immunoprecipitation assays. Toll-like receptors play a pivotal role in the induction of innate immune and inflammatory responses. Diabetes is known to be a chronic low-grade inflammatory disease. Recent studies have also demonstrated that the expression of TLR2 is increased in patients with type 1 and type 2 diabetes (Creely et al., 2007; Devaraj et al., 2008). Animal studies have shown that knockout of TLR2 in a mouse model has significant benefits in diabetes-induced cardiac dysfunction (Lei et al., 2018). Moreover, TLR2 signaling leads to the activation of the key transcription factor NF-κB. Recent studies have established that NF-κB expression is upregulated in a high glucose environment, and that inhibiting the NF-κB signaling pathway can effectively reduce cardiac oxidative stress and myocardial fibrosis, and reverse the deterioration of cardiac function (Liu et al., 2020; Youssef et al., 2021). Previous studies have demonstrated that activation of the TLR2/NF-kB pathway plays a significant role in diabetic nephropathy (Wang H. et al., 2021a).

Therefore, the goal of our study was to ascertain the functional role of NOX1 in the DCM model *in vivo* and *in vitro*. In addition, we postulated that NOX1 contributes to DCM *via* modulating the TLR2/NF-B pathway. The current study will contribute to a better understanding of the key molecular mechanisms behind DCM and is beneficial for exploring potential therapeutic targets.

## Materials and methods

### Experimental animals

Male Sprague–Dawley rats weighing 200 ± 20 g were obtained from the Animal Center of Harbin Medical University (Harbin, China). All rats were housed under controlled temperature (21 ± 1°C), humidity (55 ± 5%), and a 12 h light–dark cycle, with free access to food and water. All animal studies were approved by the Institutional Animal Care and Use Committee of Harbin Medical University (No. SYDW2019-112) and were conducted in accordance with a Laboratory Animal Care and Use Guide published by the NIH, US. After 1 week of adaptive feeding, rats were randomly divided into four groups. Two groups were fed a basal diet, while the other two groups were fed a high-fat diet (HFD; 60% fat, 20% protein, and 20% carbohydrate). Four weeks later, rats from two high-fat groups received intraperitoneal injections of STZ (40 mg/kg, Sigma, USA) in citrate buffer (pH 4.5) for three consecutive days. For other rats, only citrate buffer was administered. One week after administrating STZ, plasma glucose levels were measured using a Contour glucometer (Roche, Germany). Rats with random plasma glucose levels of over 16.7 mM were considered diabetic. Rats with substandard blood glucose were reinjected with STZ until they reached the standard. One week after the diabetic rat model was successfully established, a NOX1 specific inhibitor, ML171 (20 mg/kg, MedChemExpress, USA), was injected intraperitoneally into rats twice a week until the end of the experiment. ML171 was dissolved in DMSO, and the other two groups were given intraperitoneal injections of an equal dose of DMSO. The rats were divided into four groups based on whether or not ML171 was administered: the WT group, the WT + ML171 group, the DCM group, and the DCM + ML171 group, with *n* = 6 rats for each group. The rats were followed up for 12 weeks after the induction of diabetes for subsequent experiments.

### Echocardiography

Animals were anesthetized with isoflurane. M-mode echocardiography images of the left ventricle were used in the short-axis view to measure ejection fraction (EF), fractional shortening (FS), left ventricular end-systolic dimension (LVESD), and left ventricular end-diastolic dimension (LVEDD), among other things.

### Histopathological analysis

Tissue from the left ventricle of rats fixed in 4% paraformaldehyde was embedded in paraffin and cut into 2–4 μm sections. Sections were stained with hematoxylin and eosin (H&E) to observe myocardial histomorphology and with Masson staining to examine collagen deposition. The collagen fibers were presented in blue, in contrast to the cardiac tissues, which were stained with red. Each section was imaged using a microscope (Leica, Germany). The area of myocardial fibrosis was quantified by ImageJ.

### Immunohistochemistry

For immunohistochemical analysis, paraffin sections were dewaxed and rehydrated. After antigen retrieval, endogenous peroxidase was inactivated with 3% H_2_O_2_/methanol for 15 min. Then the sections were incubated with 5% BSA and sealed for 1 h, and then incubated overnight with anti-collagen I (Col I) antibody (1:500, Abcam, USA) at 4°C. After incubation with secondary antibodies, sections were stained with DAB and hematoxylin, and then sealed for imaging. The image was obtained using a microscope (Leica, Germany).

### Isolation and culture of cardiac fibroblasts from neonatal rats

One- to three-day-old neonatal Sprague–Dawley rats were obtained from the Experimental Animal Center of Harbin Medical University. The hearts were finely minced into small pieces (Golden et al., 2012). Heart tissues were digested with trypsin (Beyotime, China) and collagenase I (Biofroxx, Germany) into single cells. The cell suspension was mixed with a 1:2 volume of low-glucose DMEM (Hyclone, USA) containing 10% fetal bovine serum (BI, Israel) and 1% penicillin–streptomycin (Beyotime, China) to terminate digestion. Cardiac fibroblasts (CFs) were obtained by 90-min differential adherence and removal of non-adherent cells. Cell cultures were incubated in a 5% CO_2_ and 95% humidified incubator at 37°C. When the growth density of CFs was above 80%, the passage could be carried out. One or two generations of isolated fibroblasts were used for subsequent experiments. Normal glucose (NG, 5.5 mM) mediums were used in culturing CFs, while high-glucose (HG, 33 mM) culture conditions were used to mimic *in vivo* DCM.

### Treatment and transfection of cardiac fibroblasts

At around 70% confluency, CFs were treated with HG and transiently transfected with NOX1 siRNA, TLR2 siRNA, corresponding negative controls (NC), or NOX1 overexpressing pcDNA3.1-plasmid (GenePharma, China) using Lipofectamine 3,000 (Invitrogen, USA). The culture medium was replaced 6 h after transfection. The culture medium was replaced by a medium with or without ML171 (0.5 μmol/l) for 48 h. After treatment, the cells were washed twice before being used in subsequent procedures.

### Immunofluorescence

Tissue samples were quickly frozen in liquid nitrogen and stored at -80°C for later use. The tissue was sectioned with a frozen microtome, with a thickness of 5–7 μm. Frozen tissue sections were fixed in cold acetone for 15 min. CFs were fixed with 4% paraformaldehyde for 15 min. Subsequent steps were identical to those used for tissue immunofluorescence. Then 0.2% of Triton in PBS was used to penetrate the sections or cells. The 5% BSA was sealed for 1 h and then rinsed with PBS. A primary antibody α-smooth muscle actin (α-SMA) (ABclonal, China) with a dilution of 1:500 was added and incubated overnight at 4°C. On the second day, FITC- or TRITC-labeled anti-mouse secondary antibodies (1:500, ZSGB-BIO, China) were incubated at room temperature for 1 h in the dark. Nuclei were stained with DAPI (5 μg/ml, Beyotime, China) and observed under a fluorescence microscope (Leica, Germany).

### Determination of oxidative stress

Peroxide-sensitive fluorescent probe DCFH-DA (Beyotime, China) was used to detect ROS intracellular levels. After being treated with HG and/or ML171 or siRNAs, CF cells were washed with PBS three times and incubated with DCFH-DA for 30 min in the dark. The ROS levels were detected and images were obtained by fluorescence microscopy (Leica, Germany). A lipid peroxidation malondialdehyde (MDA) assay kit (Beyotime, China) was used to evaluate the MDA generation of CFs, according to the instruction manual. Absorbance at 532 nm was measured using a microplate reader (Thermo, USA).

### Real-time PCR analysis

Total RNA was extracted from CFs or cardiac tissues using TRIzol reagent (Invitrogen, USA). The total RNA was reversed by using the TransScript Reverse Transcriptase (Roche, Germany) to synthesize cDNA. The target genes were determined using the SYBR Green ROX incorporation method on a real-time PCR system (Bio-rad, USA). The relative expression levels of target genes were calculated using the relative quantitative 2^-ΔΔCT^ method. GAPDH was used as an internal control. The PCR primer sequences are shown in Table 1 **.**

**TABLE 1:** PCR primer sequences were indicated as follows (norway rat genes, 5′-3′ forward primer/reverse primer).

Gene	Sequence (5′-3′)
α-SMA-F	CCT​CTT​CCA​GCC​ATC​TTT​CAT
α-SMA-R	CGA​GAG​GAC​GTT​GTT​AGC​ATA​G
Col I-F	GTA​CAT​CAG​CCC​AAA​CCC​CA
Col I-R	GGG​ACT​TCT​TGA​GGT​TGC​CA
Col III-F	TCT​GGC​GGC​TTT​TCA​CCA​TA
Col III-R	GCA​TCC​ATC​TTG​CAG​CCT​TG
GAPDH-F	ACG​GGA​AAC​CCA​TCA​CCA​TC
GAPDH-R	ACG​ACA​TAC​TCA​GCA​CCA​GC
NF-κB-F	GGA​TGA​CAG​AGG​CGT​GTA​TAA​G
NF-κB-R	CCT​TCT​CTC​TGT​CTG​TGA​GTT​G
NOX1-F	CAC​TCC​CTT​TGC​TTC​CTT​CT
NOX1-R	GCA​CCC​GTC​TCT​CTA​CAA​ATC
TGF-β1-F	CTG​AAC​CAA​GGA​GAC​GGA​ATA​C
TGF-β1-R	GTT​TGG​GAC​TGA​TCC​CAT​TGA
TLR2-F	GAG​GGA​GCT​AGG​TAA​AGT​AGA​AAC
TLR2-R	CAC​TTT​CTC​CAG​GAG​GGA​ATA​C

### Western blot

The total protein and nuclear protein were extracted from CFs or cardiac tissues for concentration determination. Nuclear protein was isolated and extracted using nuclear protein and cytoplasmic protein extraction kits, respectively. Then, the protein samples were subjected to SDS-PAGE and electrophoretically transferred to PVDF membranes (Roche, Germany). After blocking with 5% skimmed milk powder (for non-phosphorylated proteins) or 5% BSA (for phosphorylated proteins) dissolved in TBST solution for 2 h, the PVDF membranes were incubated with primary and secondary antibodies. The specific bands were detected with enhanced ECL chemiluminescence with an imaging system (Tannon, China), and band intensities were quantified with ImageJ. Total protein levels were normalized to GAPDH, while nuclear NF-κB (n-NF-κB) (p65) levels were normalized to histone H3. Antibodies used in this study were summarized as follows: Col I (ab270993, 1:10n00, Abcam, USA), collagen III (Col III) (22734-1-AP, 1:11000 Proteintech, China), α-SMA (A17910, 1:2000, ABclonal, China), TGF-β1 (ab179695, 1:11000, Abcam, USA), NOX1 (DF8684, 1:2000, Affinity, USA), TLR2 (ab209217, 1:11000, Abcam, USA), NF-κB (p65) (8,242, 1:11000, Cell Signaling Technology, USA), IKBα (4,814, 1:11000, Cell Signaling Technology, USA), Phospho- IKBα (2,859, 1:11000, Cell Signaling Technology, USA), Histone H3 (WL0984a, 1:500, Wanleibio, Chian), and GAPDH (TA-08, 1:10000, ZSGB-BIO, China).

### Protein–protein interaction network analysis

The STRING database was used to generate the network of protein interactions. Based on experiments, the interaction network was generated with a required confidence score and “active interaction sources.”

### Statistical analysis

The data were shown as the mean ± SEM of at least three independent experiments with at least three technical replicates. The two-tailed Student’s *t*-test was used to analyze differences between the two groups. Statistical comparisons among multi-groups were evaluated by one-way ANOVA with Tukey’s *post hoc* tests. The *p* value < 0.05 was considered statistically significant.

## Results

### Expression of NOX1 was upregulated in diabetic conditions

**FIGURE 1:**
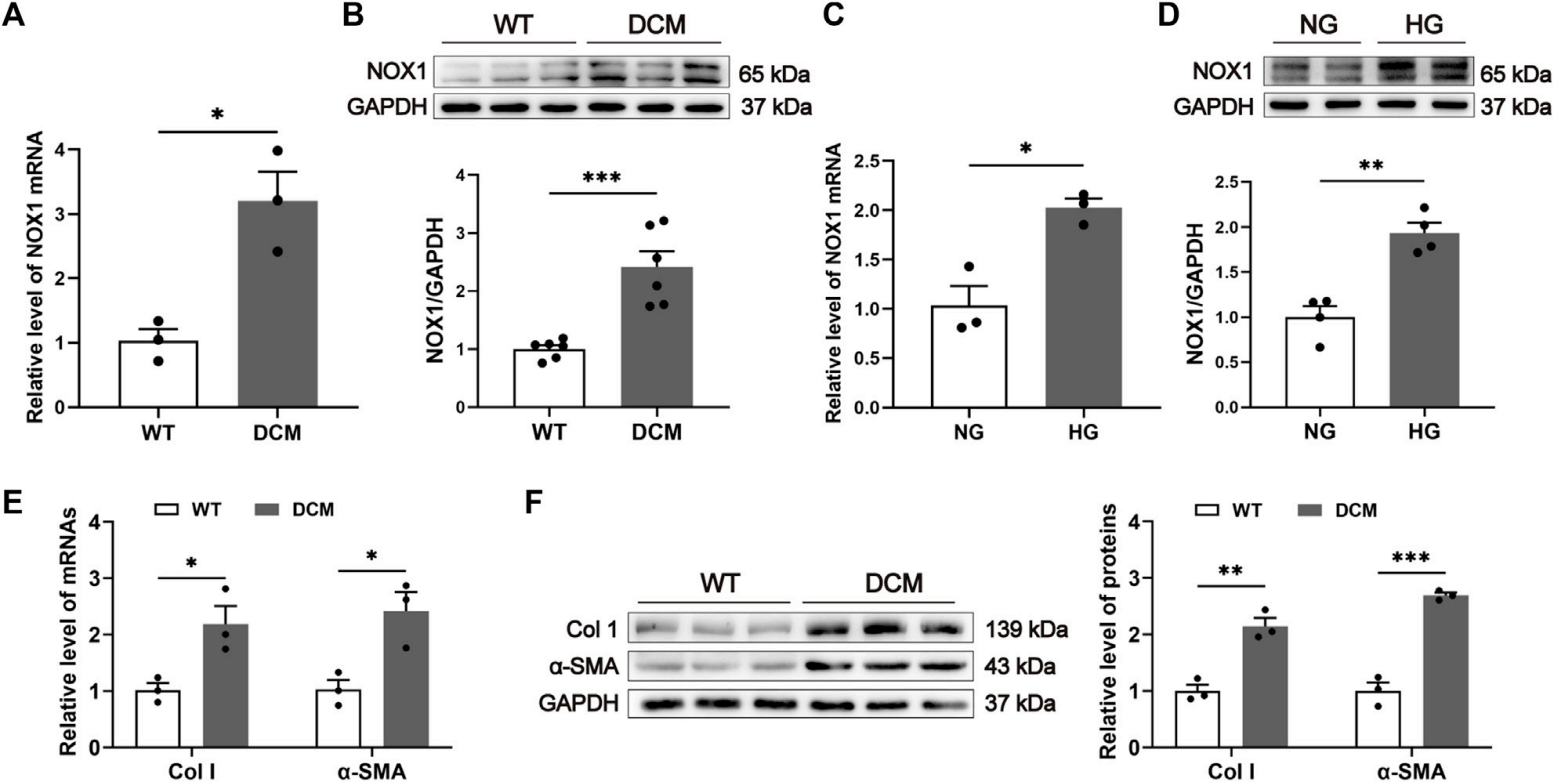
Expression of NOX1 was upregulated in DCM. **(A)** NOX1 mRNA level in cardiac tissues (*n* = 3). **(B)** Expression of NOX1 protein in heart tissues was detected and quantified (*n* = 6). **(C)** mRNA level of NOX1 in cardiac fibroblasts with or without HG treatment (*n* = 3). **(D)** Expression of NOX1 protein in cardiac fibroblasts was detected and quantified (*n* = 4). **(E)** mRNA level of Col I and α-SMA in the heart (*n* = 3). **(F)** Expression of Col I and α-SMA was detected and quantified (*n* = 3). * for *p* ≤ 0.05, ** for *p* ≤ 0.01, and *** for *p* ≤ 0.001.

A rat model of DCM by HFD and STZ injection was established in this study. The expression level of NOX1 in diabetic conditions was detected using qRT-PCR and Western blot. The results revealed that mRNA and protein expression of NOX1 were significantly upregulated in the hearts of DCM rats (Figures 1A,B). In addition, primary neonatal CFs were treated with high glucose to determine whether NOX1 expression was increased in *in vitro* diabetic conditions. Similarly, NOX1 expression was upregulated in the HG group compared with the NG group (Figures 1C,D). Furthermore, qRT-PCR and Western blot revealed increased expression of Col I and α-SMA in DCM rats (Figures 1E,F). Overall, our findings suggested that NOX1 might be linked to the pathogenesis of DCM.

### NOX1 inhibition attenuated diabetes-induced cardiac dysfunction

**FIGURE 2:**
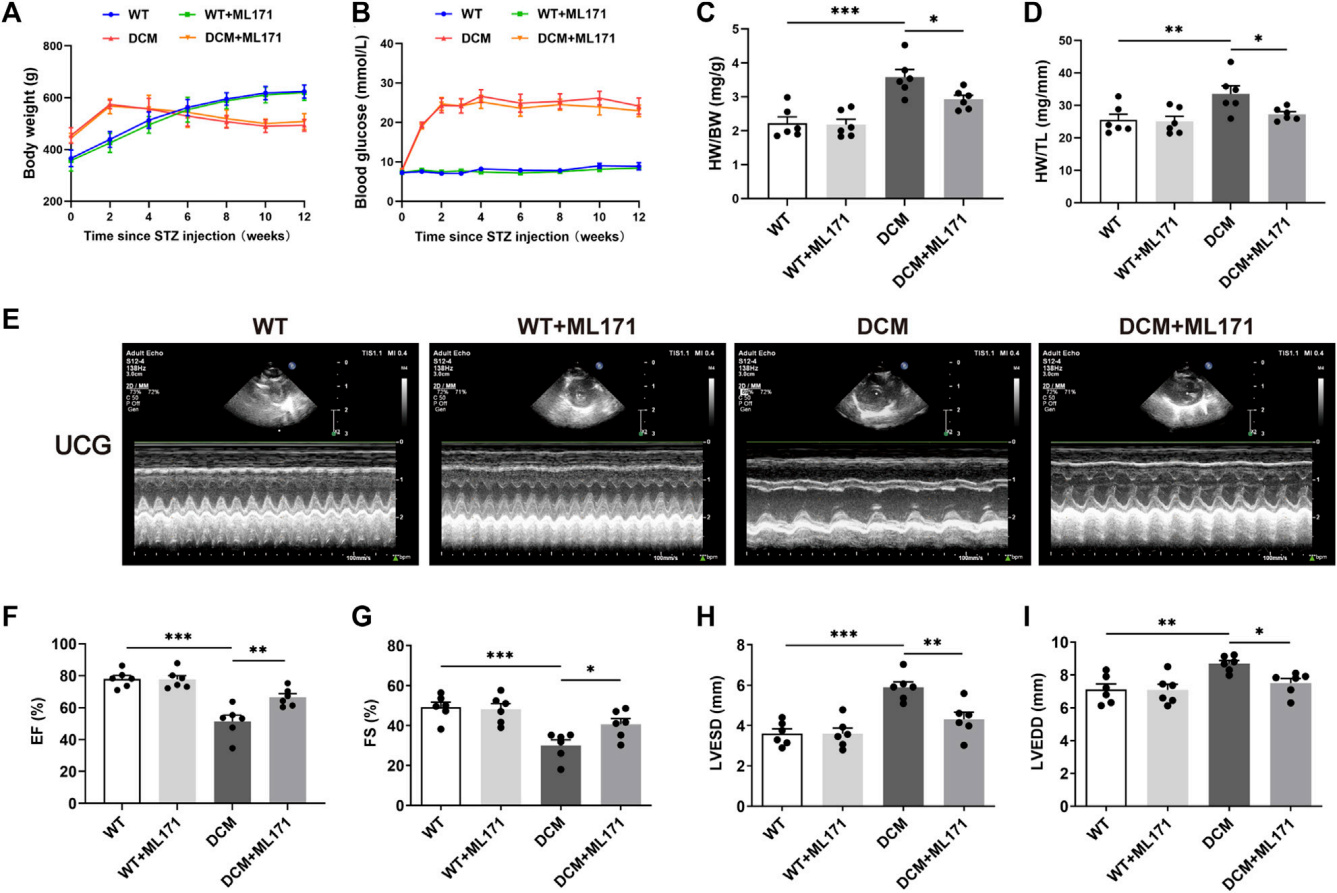
NOX1 inhibition protected against diabetes-induced cardiac dysfunction *in vivo*. **(A)** Graph summarized body weight from each experimental group. **(B)** Blood glucose concentration. **(C)** Ratio of heart weight to body weight (HW/BW). **(D)** Ratio of heart weight to tibia length (HW/TL). **(E)** Representative echocardiography image of the four groups. **(F–I)** Echocardiographic parameters (*n* = 6 per group). * for *p* ≤ 0.05, ** for *p* ≤ 0.01, and *** for *p* ≤ 0.001.

To further explore the roles of NOX1 in DCM, ML171, a specific NOX1 inhibitor, was used to test the cardiac protective function in DCM rats. qRT-PCR and Western blot analysis revealed that NOX1 expression was increased in the cardiac tissues of DCM rats compared with WT rats, while a reduction was detected in the rats treated with ML171 (Supplementary Figures S1A,B). To elucidate how NOX1 inhibition attenuated cardiac dysfunction, body weight, blood glucose, heart weight/body weight (HW/BW), heart weight/tibia length (HW/TL), and some echocardiographic parameters were measured. Body weight and blood glucose were measured throughout the study. Increased body weight and blood glucose were observed in DCM rats, but ML171 had no effect on these metabolic characteristics (Figures 2A,B). The ratios of HW/BW and HW/TL were higher in DCM rats than in WT rats, and ML171 treatment decreased this ratio (Figures 2C,D). Next, to evaluate cardiac function and cardiac remodeling, we performed echocardiography (Figure 2E). The echocardiogram of the DCM group showed that EF and FS were pointedly reduced, and LVESD and LVEDD were significantly higher than those of the WT group, while ML171 treatment led to elevations in EF and FS and reductions in LVESD and LVEDD (Figures 2F–I). Taken together, the results indicated that the inhibition of NOX1 expression preserved diabetes-induced cardiac dysfunction.

### NOX1 inhibition decreased myocardial fibrosis in diabetic myocardium

**FIGURE 3:**
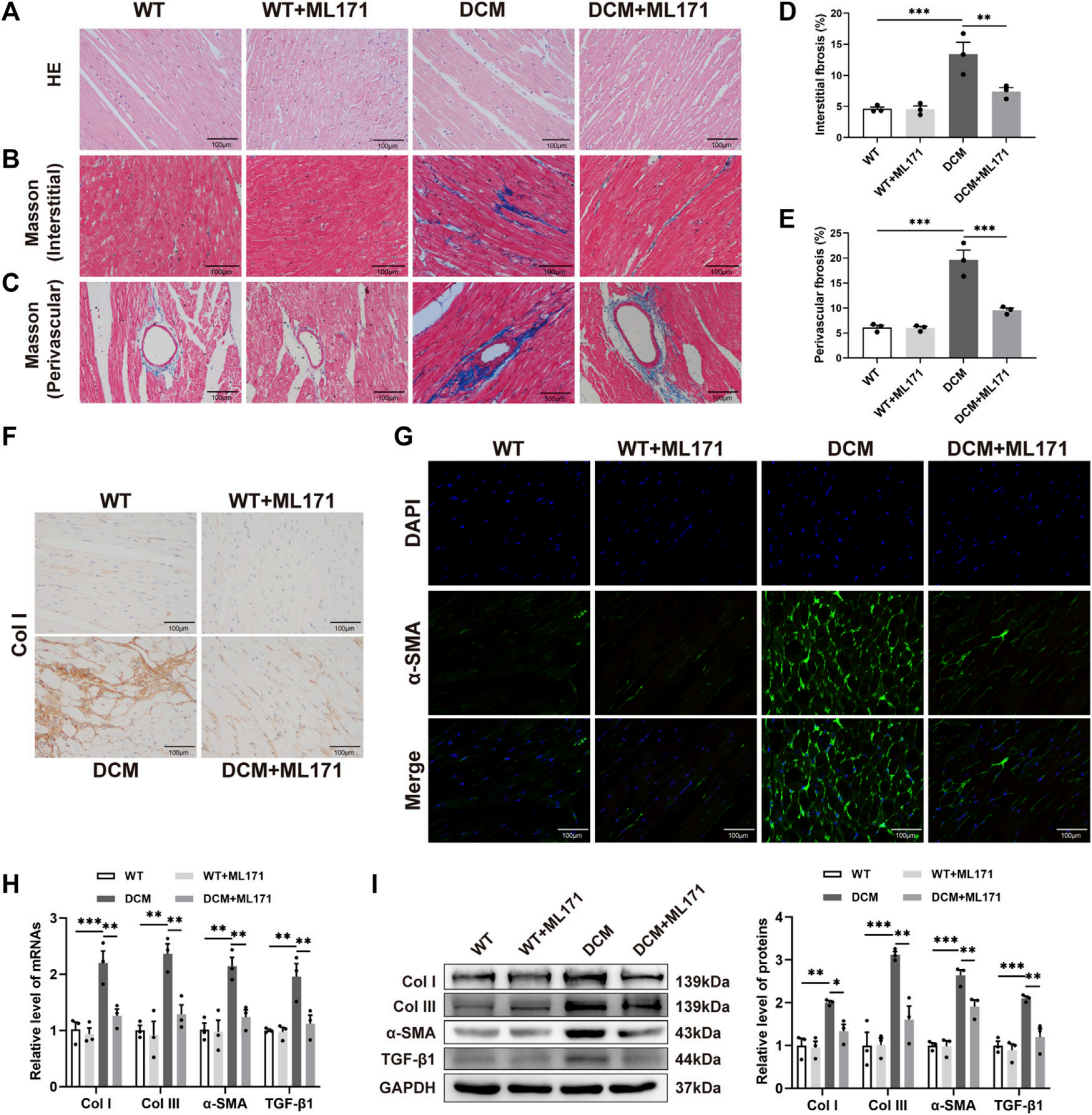
NOX1 inhibition suppressed myocardial fibrosis in DCM rats. **(A)** HE staining of the myocardium. **(B–C)** Masson’s trichrome staining of the myocardium and perivascular regions. **(D–E)** Quantification of collagen in the myocardium interstitial and perivascular regions. **(F)** Immunostaining of Col I protein expression. **(G)** Detection of expressed α-SMA protein in myocardial tissue was performed with a fluorescence microscope. **(H)** Gene expression levels of Col I, Col III, α-SMA, and TGF-β1 in each group. **(I)** Protein levels of Col I, Col III, α-SMA, and TGF-β1, and quantitative analysis in cardiac tissues (*n* = 3 per group). * for *p* ≤ 0.05, ** for *p* ≤ 0.01, and *** for *p* ≤ 0.001.

The effect of ML171 treatment on cardiac histological changes was determined by H&E staining and Masson trichrome of heart sections. Cardiac muscle fibers in the WT and ML171 groups were arranged regularly, while in the DCM group, they were disordered, the cell gap increased, and many of the fibers were collapsed in the myocardium (Figure 3A). Interstitial and perivascular fibrosis were prominent in the DCM hearts, while fibrosis levels were reduced after treatment with ML171 (Figures 3B–E). Immunohistochemical staining analysis showed that the number of Col I brown-stained positive cells increased significantly in the cardiac tissues of the DCM group, while the index was decreased in the cardiac tissues of DCM rats upon NOX1 inhibition (Figure 3F). In addition, α-SMA is a myofibroblast marker used in many previous studies. The immunofluorescent signal of α-SMA was significantly activated and increased in diabetes myocardium, while it was restored upon ML171 treatment (Figure 3G). In addition to α-SMA, Col I, Col III, and TGF-β1 are fibrogenic markers. We found increased expression of Col I, Col III, α-SMA, and TGF-β1 in the DCM group compared with the WT group. The treatment with ML171 significantly inhibited the significant upregulation of Col I, Col III, α-SMA, and TGF-β1 in the heart tissues of DCM rats (Figures 3H,I). These data indicated that the inhibition of NOX1 expression could effectively decrease myocardial fibrosis in DCM rats.

### NOX1 inhibition mitigated the TLR2/NF-κB-mediated response in diabetic myocardium

**FIGURE 4:**
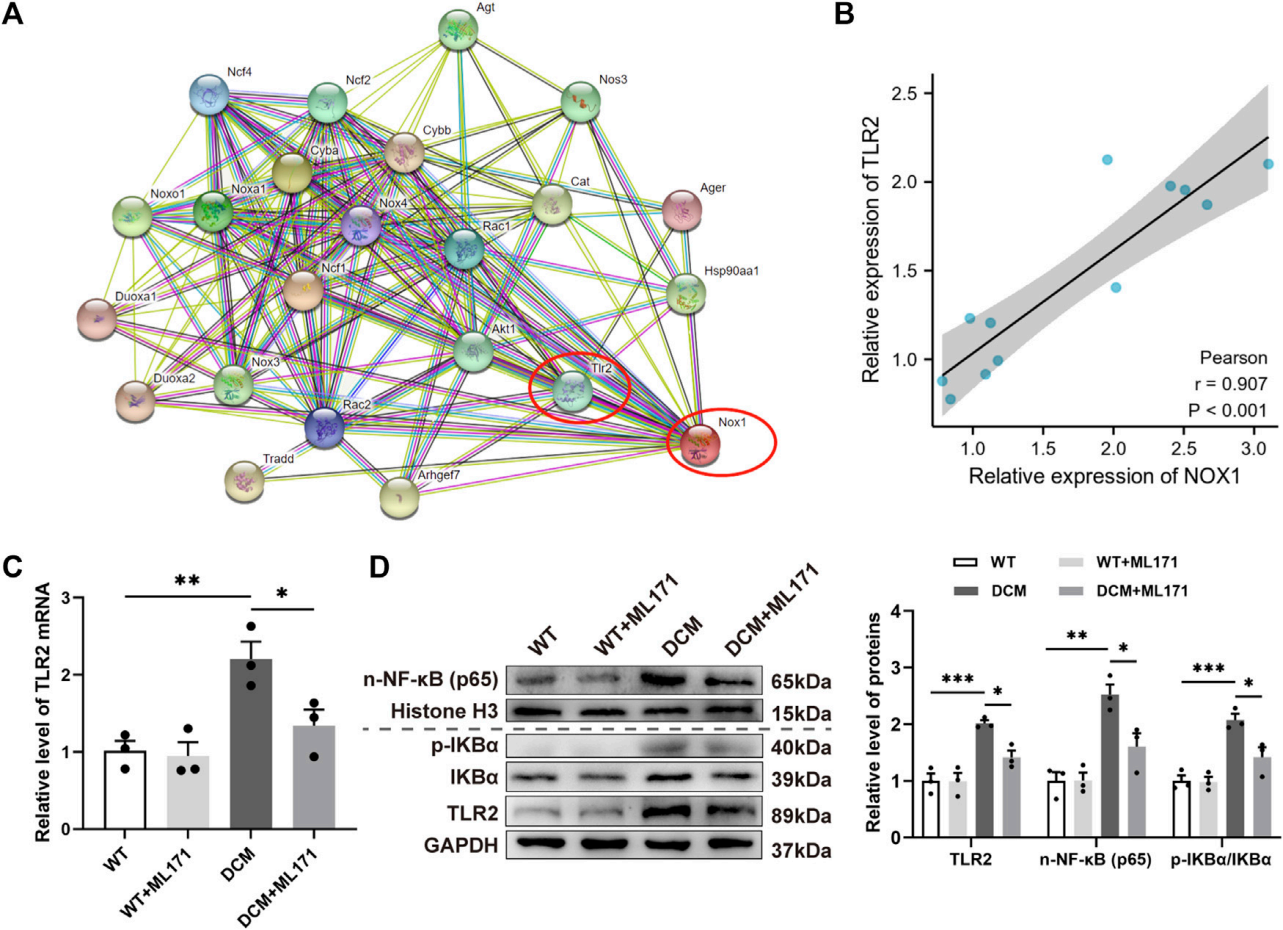
NOX1 inhibition mitigated the TLR2/NF-κB-mediated response in diabetic myocardium. **(A)** STRING database showed the potential of candidate proteins acting downstream of NOX1. **(B)** Correlation of NOX1 expression with TLR2 expression in DCM rats’ cardiac tissues was analyzed by Pearson’s correlation coefficient (*n* = 6). **(C)** Gene expression levels of TLR2. **(D)** Protein levels of TLR2, n-NF-κB (p65), and the p-IKBα/IKBα ratio and quantitative analysis (*n* = 3). * for *p* ≤ 0.05, ** for *p* ≤ 0.01, and *** for *p* ≤ 0.001.

In order to find the molecular mechanism of NOX1 regulating DCM, the STRING database was used to determine candidate proteins that may act downstream of NOX1 (Figure 4A). Previous studies have shown that TLR2 is associated with myocardial fibrosis (Wu et al., 2018). Pearson’s correlation coefficient showed that NOX1 expression was positively correlated with TLR2 expression in the cardiac tissues of DCM rats (Figure 4B).

To clarify the role of the TLR2 signaling pathway in DCM myocardial fibrosis, we investigated TLR2 and its ligand NF-κB (Dasu et al., 2010; Madonna et al., 2017) on gene transcription and protein expression levels in cardiac tissues. The expression of TLR2 mRNA increased in DCM rats, while this effect was reversed after the treatment with ML171 (Figure 4C). Western blot analysis of protein expression levels of TLR2, n-NF-κB (p65), and the p-IKBα/IKBα ratio showed that the levels of protein were significantly increased in the DCM group compared to controls, which was effectively attenuated by ML171 (Figure 4D). These results indicated that NOX1 inhibition was effective in inhibiting the TLR2/NF-κB pathway of DCM rats.

### NOX1 promoted fibrosis and oxidative stress in cardiac fibroblasts under HG conditions

**FIGURE 5:**
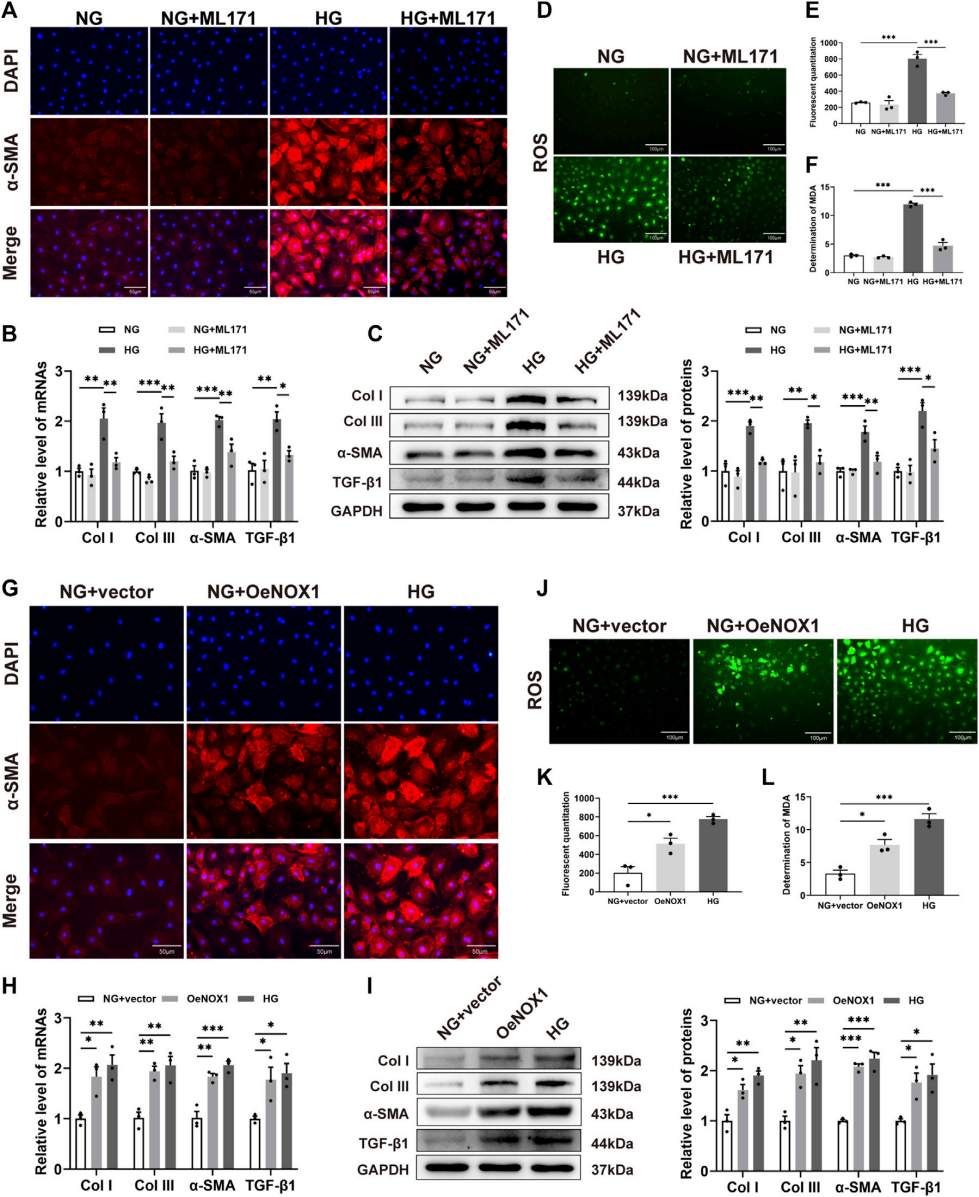
NOX1 promoting fibrosis and oxidative stress in CFs. **(A,G)** Representative image of α-SMA expression (red) in myocardial cells by immunofluorescence. The nuclei were identified by DAPI (blue). **(B,H)** Gene expression levels of Col I, Col III, α-SMA, and TGF-β1. **(C,I)** Protein levels of Col I, Col III, α-SMA, and TGF-β1, and quantitative analysis in each group. **(D,J)** Intracellular ROS level was detected by DCFH-DA staining. **(E,K)** Quantitative result of the ROS level. **(F,L)** Level of MDA in the indicated group (*n* = 3 per group). * for *p* ≤ 0.05, ** for *p* ≤ 0.01, and *** for *p* ≤ 0.001.

In order to verify the effects of NOX1 upon HG-induced CFs *in vitro*, ML171 treatment, NOX1 siRNA, and NOX1 overexpression plasmids were used in CFs. NOX1 inhibition or transfection efficiency is shown in Supplementary Figures S1C–H. We first examined the expression and localization of α-SMA proteins in CFs after HG treatment by immunofluorescence. As depicted in Figure 5A, the fluorescence intensity of α-SMA significantly increased in the HG group in comparison with the NG group, while it decreased evidently in the HG + ML171 group. Then, we confirmed that high glucose promoted the production of Col I, Col III, α-SMA, and TGF-β1 in CFs at both the mRNA and protein levels. In contrast, ML171 mitigated the stimulating effects of HG on CFs (Figures 5B,C).

Oxidative stress plays an important role in diabetic cardiomyopathy. The levels of ROS and MDA were examined, which reflected the degree of myocardial oxidative stress. DCFH-DA staining revealed that the level of ROS in HG-induced CFs was significantly higher than that in NG-cultured CFs. However, ML171 alleviated the level of ROS caused by HG (Figures 5D,E).The changes in MDA content in CFs were similar to those of ROS (Figure 5F).

To further investigate the function of NOX1, CFs were transfected with NOX1 siRNA or negative siRNA and then stimulated with HG. These findings were similar to those obtained with ML171 administration (Supplementary Figure S2). We then tested whether the upregulation of NOX1 was a causal factor for collagen and ROS production in CFs. In particular, immunofluorescence revealed increased α-SMA expression in both HG treatment and overexpression of NOX1 *in vitro* (Figure 5G). Here, we also observed the increase of Col I, Col III, α-SMA, and TGF-β1 in the two groups compared to the NG group (Figures 5H,I). DCFH-DA staining and MDA determination showed that the overexpression of NOX1 could induce the activation of ROS (Figures 5J–L). It was shown by DCFH-DA staining and MDA measurements that the overexpression of NOX1 could induce increased ROS production (Figures 5J–L).

Therefore, the abovementioned results showed that NOX1 promoted fibrosis and oxidative stress in HG-induced CFs.

### NOX1 regulated the activation of TLR2/NF-κB in HG-induced cardiac fibroblasts

**FIGURE 6:**
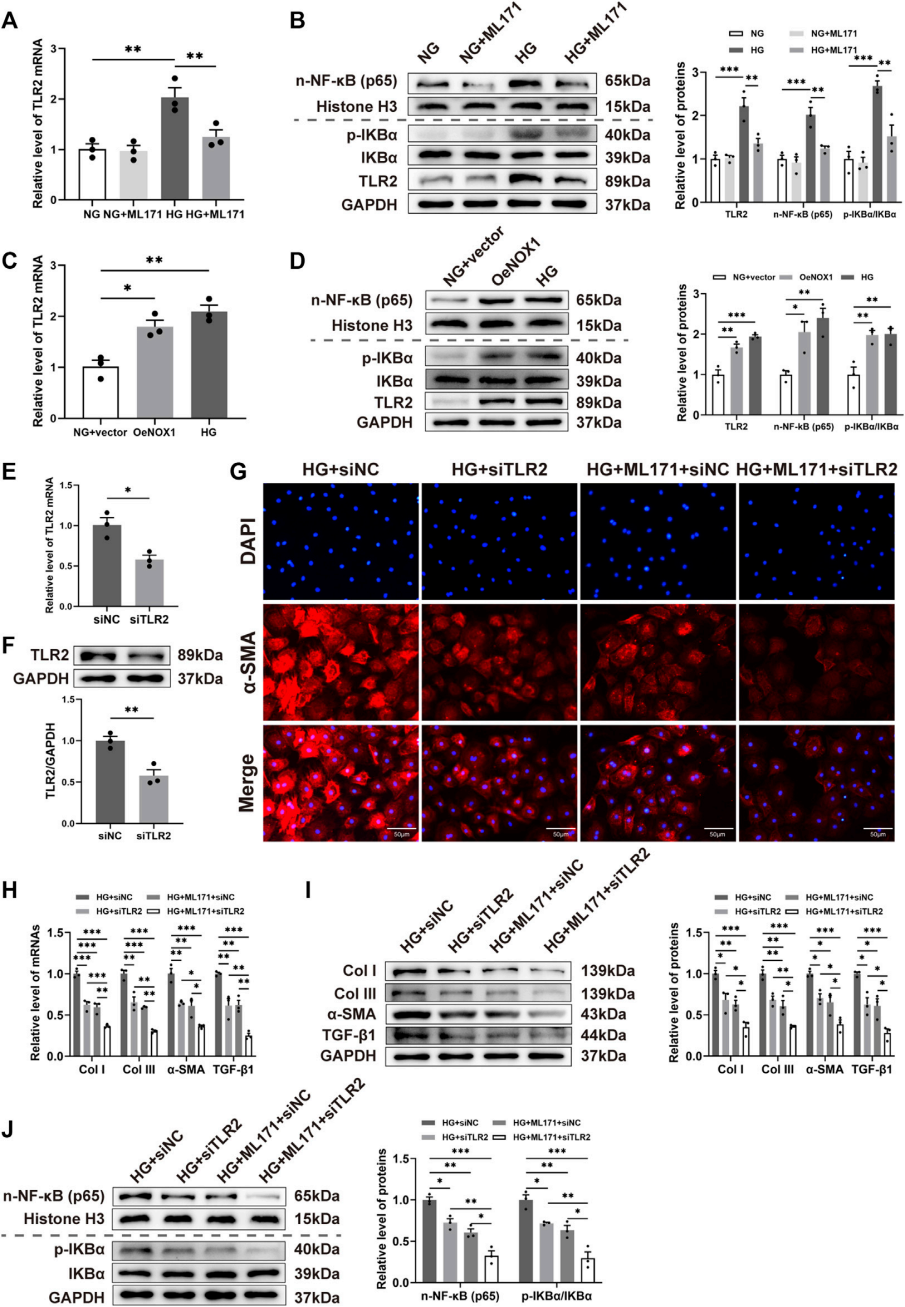
NOX1 regulated the activation of the TLR2/NF-κB pathway in the HG-induced CFs. **(A,C,E)** Gene expression levels of TLR2. **(B,D,F,J)** Protein levels of TLR2, n-NF-κB (p65), and the p-IKBα/IKBα ratio and quantitative analysis. **(G)** Representative image of α-SMA expression (red) in myocardial cells by immunofluorescence. The nuclei were identified by DAPI (blue). **(H)** Gene expression levels of Col I, Col III, α-SMA, and TGF-β1. **(I)** Protein levels of Col I, Col III, α-SMA, and TGF-β1, and quantitative analysis in each group (*n* = 3 per group). * for *p* ≤ 0.05, ** for *p* ≤ 0.01, and *** for *p* ≤ 0.001.

In addition, we performed the validation of the TLR2/NF-κB pathway *in vitro*. The results of qRT-PCR revealed that ML171 could inhibit the level of TLR2 mRNA in CFs induced by HG (Figure 6A). We also found that the levels of TLR2, n-NF-κB (p65) proteins, and the p-IKBα/IKBα ratio were significantly elevated in the HG-treatment group, while the administration of ML171 was decreased following HG treatment (Figure 6B). Here, we observed that both the HG treatment and overexpression of NOX1 *in vitro* activated the TLR2/NF-κB pathway, which is further confirmed by the increased mRNA level of TLR2 and protein levels of TLR2, n-NF-κB (p65), and the p-IKBα/IKBα ratio (Figures 6C,D).

To investigate the role of TLR2 during HG-induced CFs, we knocked down TLR2 using appropriate siRNA (Figures 6E,F). Immunofluorescence revealed decreased α-SMA expression in either the ML171 treatment or TLR2 siRNA group (Figure 6G). Our results suggested that TLR2 deficiency or ML171 administration prevented the mRNA and protein levels of Col I, Col III, and α-SMA, and TGF-β1 increased in HG-induced CFs (Figures 6H,I). We also observed alterations in n-NF-κB (p65) and the p-IKBα/IKBα ratio in cells upon TLR2 silencing or administering ML171 after HG stimulation (Figure 6J). Interestingly, these differences became significantly more pronounced after TLR2 knockdown and combination treatment with ML171. Our results showed that TLR2 is a driving factor for the activation of cardiac fibroblasts. Thus, we concluded that NOX1 promoted fibrosis *via* activating the TLR2/NF-κB pathway in the HG-treated CFs.

## Discussion

As a result of our research, we have demonstrated that NOX1 is a critical molecule in the development of DCM. The inhibition of NOX1 expression alleviated cardiac dysfunction, reduced myocardial fibrosis, and inhibited oxidative stress in DCM. The upregulation of NOX1 expression promoted myocardial fibrosis through the activation of the TLR2/NF-κB pathway. To our knowledge, this is the first study to demonstrate the involvement of NOX1 during myocardial fibrosis in DCM, thereby adding a new mechanistic insight and potential therapeutic target for DCM.

DCM is a disorder of the heart muscle caused by diabetes, and its underlying mechanism of pathogenesis is complex and unclear. Hyperglycemia, hyperinsulinemia, dyslipidemia, oxidative stress, inflammation, and insulin resistance all contribute to cardiovascular complications in diabetic patients (Boudina and Abel, 2010; Palomer et al., 2018; Ritchie and Abel, 2020). DCM is aggravated by increased ROS production in the diabetic heart (Boudina and Abel, 2007; Boudina et al., 2009). NOX1, a member of the NOX family, is one of the major sources of ROS in cardiac tissue (Lassegue et al., 2012). Multiple studies have shown that deficiency or overexpression of NOX1 has revealed its role in the pathogenesis of cardiovascular diseases, including atherosclerosis, restenosis, hypertension, and ischemia/reperfusion injury (Gimenez et al., 2016). NOX1 is an important factor in mediating oxidative stress and promoting fibrosis in other tissues (Paik et al., 2011; Zhu et al., 2015; Jung et al., 2020). In addition to NOX1, two more NOX isoforms, NOX2 and NOX4, are mainly expressed in cardiac tissues. Among them, NOX2 and NOX4 have been shown to contribute to DCM (Liu et al., 2012). The role of NOX4 in DCM has been well explained by Maalouf et al. (2012), who found that inhibiting the direct heart effect of NOX4 reduced the production of ROS in the heart caused by diabetes, which led to better mechanical function. Studies have proven that NOX1 is a potential therapeutic target in diabetes mellitus-related vasculopathies (Gray et al., 2013). So we wondered about the potential mechanism of NOX1’s function on DCM.

The diabetic rat model was created using HFD combined with STZ, and it showed cytological and metabolic alterations comparable to those reported in human cardiomyopathies (Reed et al., 2000; Bugger and Abel, 2009). Subsequently, we discovered that the expression of NOX1 was upregulated in DCM rats. This observation was also confirmed by *in vitro* experimental data. Animal studies of type 2 diabetes also showed that NOX activity was upregulated in the myocardium and vascular tissues. An increase in NOX activity in the myocardium and vascular tissues in type 2 diabetic animal models has also been observed (Inoguchi et al., 2003; Matsushima et al., 2009), which confirmed our findings. In order to further explore the mechanism of NOX1 regulation of DCM, ML171 was injected intraperitoneally into rats. ML171, a cell-active, nanomolar, and specific NOX1 inhibitor, has potently inhibited ROS creation through the NOX1-dependent ROS generation, but has only a little effect on other NOX isoforms (Gianni et al., 2010). The inhibitory effect of ML171 on NOX1 expression was also confirmed in our experiment. In our study, DCM rats showed deterioration of cardiac function and cardiac hypertrophy, specifically reflected by the elevations in EF and FS, and reductions in LVESD and LVEDD, and the increase of HW/BW and HW/TL ratio, which were in line with earlier research (Li et al., 2019). Furthermore, ML171 reversed these alterations, demonstrating a positive effect on diabetic cardiomyopathy dysfunction and hypertrophy. Metabolic indicators like weight and blood glucose, on the other hand, were unaffected by ML171. We speculated that NOX1 promoted diabetic cardiomyopathy independent of blood glucose levels. This opens up a whole new field of treatment for people with diabetes, in addition to hypoglycemic drug therapy.

Myocardial fibrosis is an important pathological feature of DCM. We also confirmed that fibrosis plays a role in the development of diabetic cardiomyopathy, as evidenced by the accumulation of collagen in the myocardial interstitial and perivascular region in the heart tissues of DCM rats, as well as the increase in transcription and protein expression of Col1, Col3, α-SMA, and TGF-β1 both *in vivo* and *in vitro*, findings that were in line with previous studies (Huynh et al., 2014; Wang Y. H. et al., 2021b). The abovementioned effects were significantly attenuated with NOX1 inhibition, and further enhanced with NOX1 overexpression. In addition, the profibrotic effect of NOX1 has been previously demonstrated in other organs (Paik et al., 2011; Wang F. T. et al., 2019a). Previous studies confirmed that the upregulation of NOX1/NADPH oxidase promoted cardiac dysfunction and fibrosis after drug-induced myocardial injury (Iwata et al., 2018). These were consistent with what we discovered. Here, we also discovered that oxidative stress was elevated substantially in the HG-treated CFs. According to the reports, hyperglycemia-induced oxidative stress could contribute to the generation of ROS, an event that is more susceptible to the development of diabetes complications (Brownlee, 2001; Lan et al., 2013). Our data were consistent with these findings. Simultaneously, we also observed that ML171 can dramatically reduce the level of ROS in CFs, namely, by reducing NOX1 activity. In this study, NOX1 was found to aggravate the progression of DCM by promoting myocardial fibrosis and oxidative stress induced by high glucose.

TLR2 is a pattern recognition receptor, which is involved in the pathological process of various heart diseases (Shishido et al., 2003; Spurthi et al., 2018). The STRING database indicated that TLR2 may act on one of the downstream candidate proteins of NOX1. Lee et al. (2013)reported that TLR2 had an interaction with NOX1 corroborating our conjecture, based on evidence from using yeast two-hybrid and co-immunoprecipitation assays. Moreover, NOX1 was found to regulate the expression of TLR2 in DCM rats and HG-induced CFs. In our study, the expression of TLR2 was elevated in diabetes conditions. The expression of TLR2 decreased when NOX1 was inhibited and increased when NOX1 was overexpressed. According to these findings, TLR2 was found to play a significant role in NOX1-induced fibrosis in DCM. Previous studies have confirmed that TLR2 is involved in the occurrence of myocardial fibrosis (Wu et al., 2018), which was also demonstrated in our study. We discovered that the expression of fibrosis factors was reduced after TLR2 siRNA transfected in HG-induced CFs. Combined administration of ML171 and TLR2 siRNA caused greater effects than ML171 or TLR2 siRNA alone. These results indicated that TLR2 also played an important role in myocardial fibrosis in diabetic cardiomyopathy. Different from our results, the study by Lee et al. showed that TLR2 could regulate the expression of NOX1. This phenomenon may be caused by the interaction between NOX1 and TLR2, which has been mentioned previously. However, whether there is an interaction between NOX1 and TLR2 in DCM remains to be further clarified.

**FIGURE 7:**
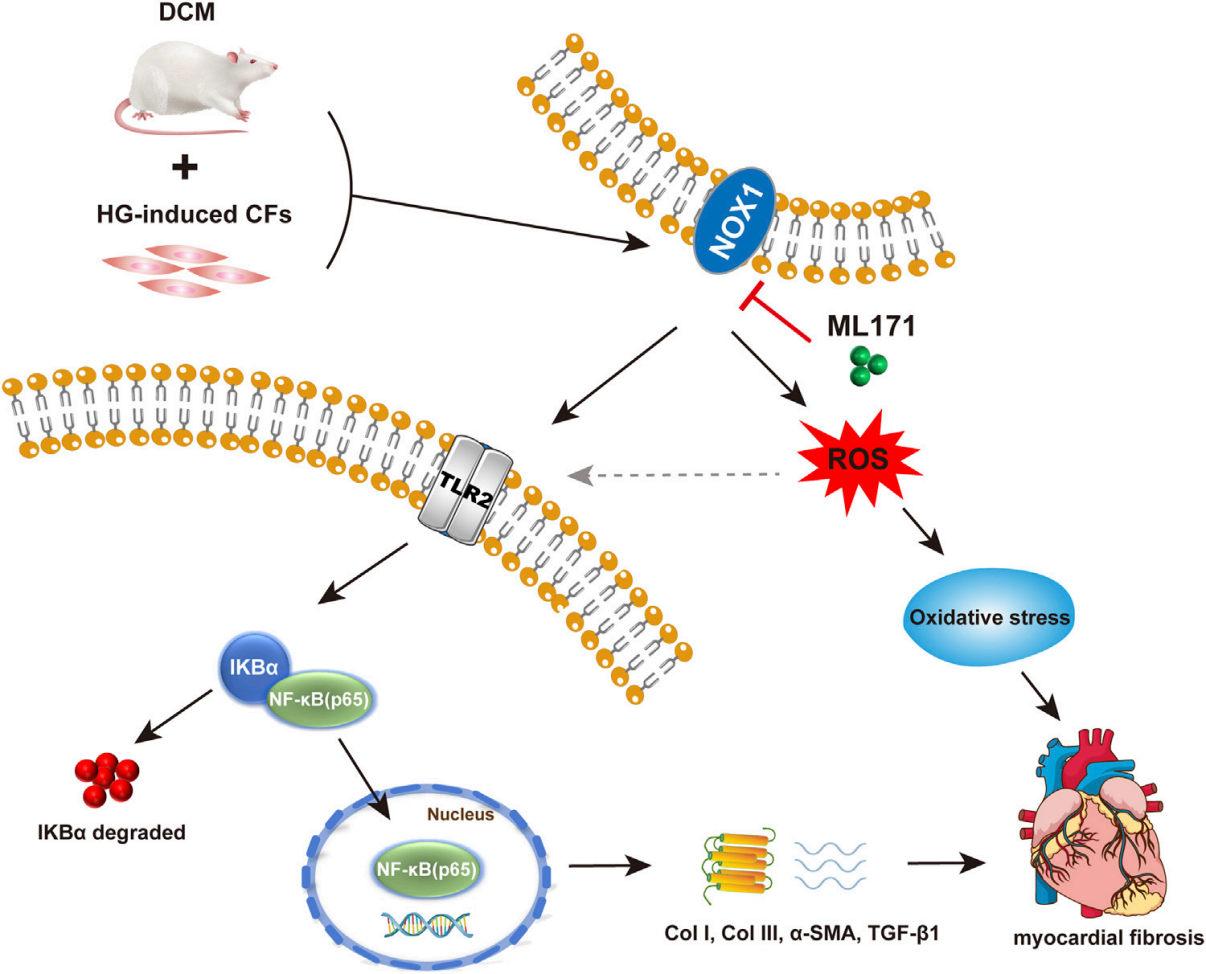
Mechanism of NOX1 promoting myocardial fibrosis *via* activating the TLR2/NF-κB pathway in diabetic cardiomyopathy.

As we all know, TLR2 has the ability to regulate the activation of NF-κB (Lim and Staudt, 2013). The activation of NF-κB is characterized by IKB kinase and leads to IKBα phosphorylation and degradation, allowing NF-κB to translocate to the nucleus. In our results, the protein Western blot showed increased expression of n-NF-κB (p65) and the p-IKBα/IKBα ratio, which was the evidence of NF-κB activation in diabetic conditions. Previous research has confirmed this (Peterson et al., 2018). Wang W. Z. et al. (2019b)found that anti-TLR2 antibody inhibits NF-κB activation and reduces cardiac damage in high-fat-feeding rats. Our study also confirmed that TLR2 regulated the activation of NF-κB. Taken together, our study showed that NOX1 inhibition reduced the activity of the TLR2/NF-κB pathway, improving myocardial fibrosis and cardiac dysfunction in DCM rats. *In vitro* experiments have shown that NOX1 promoted oxidative stress in HG-induced CFs, and promoted fibrosis by activating the TLR2/NF-κB pathway, which may be regulated by the ROS level (Figure 7). In brief, these data suggest that NOX1 promotes myocardial fibrosis in diabetic cardiomyopathy by activating the TLR2/NF-κB pathway.

Although ML171 has a significant inhibitory effect on NOX1 expression, myocardial specificity is not strong, which can be further verified by gene knockout mice. The ROS level was not verified *in vivo* in this study, which can be supplemented later. It is not clear whether there is a direct interaction between NOX1 and TLR2, which can be verified in the future.

Despite several limitations, our data suggest that NOX1 contributes to the exacerbation of diabetic cardiomyopathy by increasing fibrosis and oxidative stress. As a result of these findings, NOX1 appears to be a promising therapeutic target for diabetic cardiomyopathy.

## Conclusion

In summary, we demonstrated that NOX1 promoted myocardial fibrosis and cardiac dysfunction *via* activating the TLR2/NF-κB pathway in diabetic cardiomyopathy. Overall, our new findings suggest that NOX1 may have important translational value in the treatment of diabetic cardiomyopathy. The possible role of ML171, a NOX1 specific inhibitor, is also highlighted in this context.

## Data Availability

The original contributions presented in the study are included in the article/Supplementary Material; further inquiries can be directed to the corresponding author.
