# Supplementary material for: NOX1 promotes myocardial fibrosis and cardiac dysfunction via activating the TLR2/NF-κB pathway in diabetic cardiomyopathy
Source: Front Pharmacol. 2022 Sep 26;13:928762. doi: 10.3389/fphar.2022.928762 (PMC9549956; doi:10.3389/fphar.2022.928762)
Supplement: Supplementary file 1 [file DataSheet1.DOCX]

Supplementary Material

## Supplementary Figures


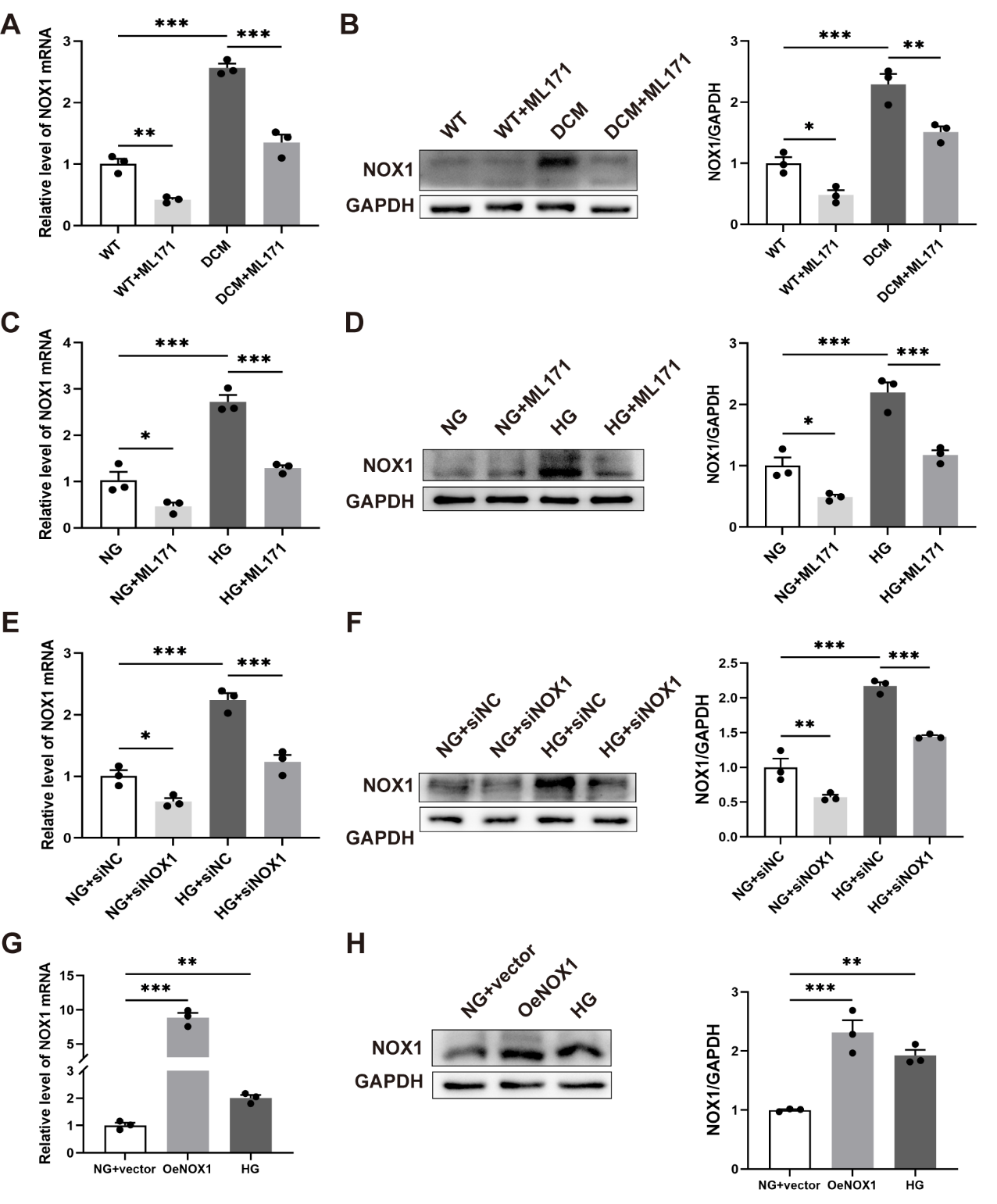


**Supplementary Figure 1.** NOX1 inhibition or transfection efficiency in different groups. **(A,C,** **E,G)** Gene expression levels of NOX1. **(B,D,F,H)** Protein levels of TLR2 and quantitative analysis.


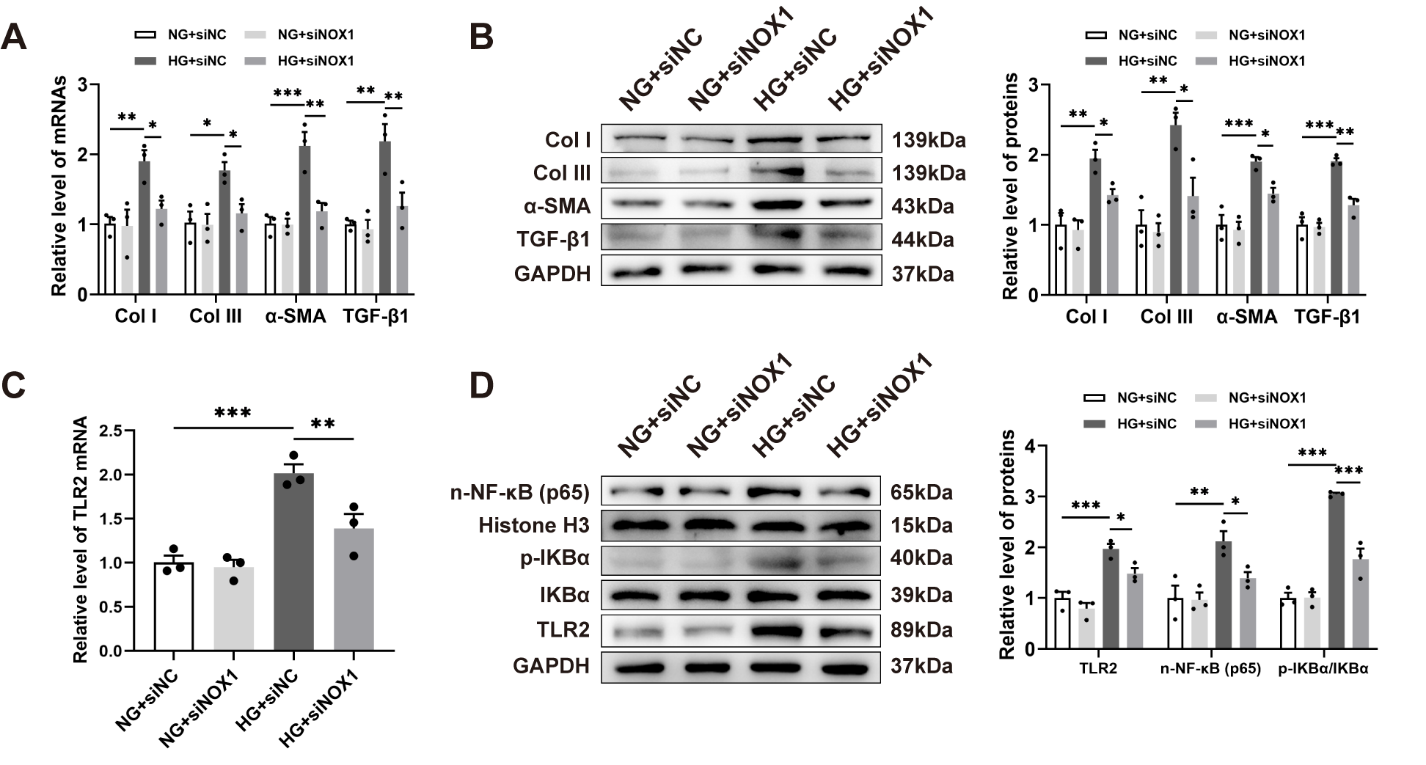


**Supplementary Figure 2.** NOX1 silencing alleviated fibrosis by inhibiting the TLR2/NF-κB pathway in HG-induced CFs. **(A)** Gene expression levels of Col I, Col III, α-SMA and TGF-β1. **(B)** Protein levels of Col I, Col III, α-SMA and TGF-β1 and quantitative analysis in each group. **(C)** Gene expression levels of TLR2. **(D)** Protein levels of TLR2, n-NF-κB (p65), and the p-IKBα/IKBα ratio and quantitative analysis.
